# Characteristics of HIV target CD4 T cells collected using different sampling methods from the genital tract of HIV seronegative women

**DOI:** 10.1371/journal.pone.0178193

**Published:** 2017-06-01

**Authors:** Smita S. Iyer, Michael J. Sabula, C. Christina Mehta, Lisa B. Haddad, Nakita L. Brown, Rama R. Amara, Igho Ofotokun, Anandi N. Sheth

**Affiliations:** 1 Division of Microbiology and Immunology, Emory Vaccine Center, Yerkes National Primate Research Center, Emory University School of Medicine, Atlanta, Georgia, United States of America; 2 Department of Biostatistics, Emory University Rollins School of Public Health, Atlanta, Georgia, United States of America; 3 Department of Gynecology and Obstetrics, Emory University School of Medicine, Atlanta, Georgia, United States of America; 4 Division of Infectious Diseases, Department of Medicine, Emory University School of Medicine, Atlanta, Georgia, United States of America; 5 Department of Microbiology and Immunology, Emory University School of Medicine, Atlanta, Georgia, United States of America; 6 Grady Infectious Diseases Program, Grady Health System, Atlanta, Georgia, United States of America; Centers for Disease Control and Prevention, UNITED STATES

## Abstract

**Background:**

Understanding the immune profile of CD4 T cells, the primary targets for HIV, in the female genital tract (FGT) is critical for evaluating and developing effective biomedical HIV prevention strategies in women. However, longitudinal investigation of HIV susceptibility markers expressed by FGT CD4 T cells has been hindered by low cellular yield and risk of sampling-associated trauma. We investigated three minimally invasive FGT sampling methods to characterize and compare CD4 T cell yield and phenotype with the goal of establishing feasible sampling strategies for immune profiling of mucosal CD4 T cells.

**Methods and results:**

FGT samples were collected bimonthly from 12 healthy HIV negative women of reproductive age in the following order: 1) Cervicovaginal lavage (CVL), 2) two sequential endocervical flocked swabs (FS), and 3) two sequential endocervical cytobrushes (CB1, CB2). Cells were isolated and phentoyped via flow cytometry. CD4 T cell recovery was highest from each individual CB compared to either CVL or FS (p < 0.0001). The majority of CD4 T cells within the FGT, regardless of sampling method, expressed CCR5 relative to peripheral blood (p < 0.01). Within the CB, CCR5^+^ CD4 T cells expressed significantly higher levels of α_4_β_7_, CD69, and low levels of CD27 relative to CCR5^-^ CD4 T cells (all p < 0.001). We also identified CD4 Treg lineage cells expressing CCR5 among CB samples.

**Conclusions:**

Using three different mucosal sampling methods collected longitudinally we demonstrate that CD4 T cells within the FGT express CCR5 and α_4_β_7_ and are highly activated, attributes which could act in concert to facilitate HIV acquisition. FS and CB sampling methods can allow for investigation of strategies to reduce HIV target cells in the FGT and could inform the design and interpretation microbicide and vaccine studies in women.

## Introduction

The majority of HIV infections by heterosexual transmission occur in adult and adolescent women across the female genital tract (FGT) mucosa [1, 2]. Understanding factors contributing to HIV acquisition at the main site of infection in women is critical for developing effective biomedical HIV prevention interventions such as pre-exposure prophylaxis (PrEP) strategies, microbicides, and vaccines, as well as for evaluating factors that may alter HIV acquisition risk, such as hormonal contraception.

Within the FGT mucosa, the number and type of cellular targets, primarily CD4^+^ T cells expressing the cell surface receptor C-C chemokine receptor type 5 (CCR5, the primary HIV co-receptor), predicts susceptibility to HIV infection [3, 4]. In the FGT mucosa, these markers are increased compared to the blood and penile mucosa [5–7], potentially explaining women’s increased risk of HIV acquisition during unprotected vaginal sex compared to men [8]. In addition to HIV co-receptor expression, CD4 T cells are heterogeneous with regards to their HIV susceptibility, and certain T cell phenotypes such as activated T cells [9] and cells expressing mucosal trafficking markers (such as α_4_β_7,_ a heterodimeric integrin receptor involved in T cell migration into the gut associated lymphoid tissue) [7] have been associated with HIV acquisition in experimental models [10, 11] and may be over-expressed in the FGT compared to blood.

Despite knowledge of these cellular markers of HIV susceptibility, inter and intra-individual variability in these markers is not well described but is critical in the design and interpretation HIV biomedical prevention studies. Of note, longitudinal studies of these markers have been hindered by challenges associated with mucosal sampling, in which trauma induced by invasive sampling (such as biopsy collection) may affect subsequent mucosal immune characterization. Furthermore, while HIV target CD4 T cells in the systemic, lymphoid, rectal, and female genital mucosal compartments have been frequently studied, detailed knowledge about specific CD4 T cell subsets, phenotypes, and functional characteristics likely to facilitate HIV acquisition in the mucosa remains limited, partially due to limited cellular yield to conduct more in-depth phenotypic and functional analyses. Recently, McKinnon *et al* compared absolute yields of mononuclear leukocyte subpopulations from genital leukocytes obtained by cervicovaginal lavage (CVL), endocervical cytobrushes, and cervical biopsy and illustrated that cell yields for all leukocyte populations tested from two serially collected cytobrushes were significantly higher than CVL and comparable to those obtained from a single cervical biopsy [12]. However, T cell yield was significantly lower in cytobrush samples compared to biopsy, raising the question of whether cytobrush samples are suitable for in-depth characterizations of CD4 T cells within the FGT which may require high cellular yields for assays.

Because characterization of FGT-derived CD4 T cells using minimally-invasive sampling methods is important for longitudinal evaluation of these cells during biomedical HIV prevention studies, we compared CD4 T cell yields and in-depth T cell subset phenotypes from cells collected longitudinally using three different non-biopsy, minimally-invasive genital tract sampling methods: CVL, two endocervical flocked swabs (FS), and each of two sequential endocervical cytobrush (CB) collections. Additionally, we determined whether two consecutive cytobrushes provide sufficient T cell yields for in-depth phenotypic characterization of FGT CD4 T cell subsets.

## Methods

### Study design and participants

We recruited HIV seronegative women between 18–44 years old with an intact uterus and cervix who were either enrolled in the Atlanta site of the Women’s Interagency HIV Study (WIHS) or who met risk factors for HIV acquisition as per enrollment criteria for the cohort (a. injection drug use or use of crack, cocaine, heroin, or methamphetamine; b. diagnosed with a sexually transmitted infection; c. unprotected sex with 3 or more men; d. having sex for drugs, money, or shelter; e. sex with a known HIV-positive man; f. having a partner meeting any of the preceding criteria). Most of the recruited participants were African American, who are disproportionately affected by HIV in the Southern US, and are representative of the demographic of the Atlanta WIHS cohort. Negative HIV serostatus was confirmed at the time of screening using an FDA-approved HIV antibody test. Women were excluded if they were pregnant, currently using systemic hormonal contraceptives, had a symptomatic vaginal infection or genital ulcer disease at screening or treatment for vaginal infection in the preceding two weeks, an active malignancy, were using immunosuppressive medications, had surgery within the preceding 2 months, or had a cervical procedure within the previous 30 days. This protocol was approved by the Institutional Review Board at Emory University and the Grady Research Oversight Committee. Written informed consent was obtained from all participants.

### Specimen collection

We collected paired blood and cervicovaginal samples during the follicular (between 7–10 days from the start of the previous menstrual cycle) and luteal (between 21–25 days from the start of the previous menstrual cycle) phases for up to five consecutive visits. Sexual, reproductive, medication, and genitourinary symptom histories were collected at each study visit. Cervicovaginal specimens were collected by the same clinician for all participants during speculum pelvic examination in the following distal to proximal order to reduce any potential effect of one collection method on the subsequent[13]: 1) CVL with 10ml phosphate buffered saline (PBS) directed toward the cervical os i.e., the opening in the center of the ectocervix, and vaginal walls x 1 minute lavage and placed in 5mL human AB serum (Atlanta Biologicals), 2) two sequential endocervical FS (COPAN Innovation, Italy) placed in 20mL complete media (RPMI -1640, penicillin-streptomycin, L-glutamine (Gibco) with 10% heat inactivated fetal bovine serum [FBS]), 3) two sequential endocervical CB collected in 20mL complete media each. Specimens were collected and immediately transported on ice for real-time processing and immune phenotyping. Presence of visible blood contamination was assessed in all FGT specimens at the time of sample collection, recorded on a scale of 1–10 at the time of processing for FS and CB; CVL was tested for blood and leukocytes using Mutistix 8SG urinalysis strips (Siemens Healthcare, Los Angeles, CA) and for semen using the ABACard p30 antigen detection test (Abacus Diagnostics, West Hill, CA). Vaginal swabs were tested for sexually transmitted infections (STIs) including *Neisseria gonorrhea*, *Chlamydia trachomatis*, *Trichomonas vaginalis*, and HSV-1/2 DNA via multiplex PCR in a CLIA-certified laboratory. Blood was collected in 8 mL-sodium citrate-containing CPT vacutainer tubes (BD, Franklin Lakes, NJ).

### Sample processing

All samples were processed within three hours of collection. CVLs and CBs were processed at 4°C as described by McKinnon et al [12]. Briefly, CVL samples were centrifuged at 1500 rpm for 5min, the cell pellet was resuspended in 10 ml complete media and the cell suspension was strained through 100 μm cell strainer up to 3 times, washed once with 10ml complete medium and resuspended in complete medium at 20 million cells/ml. Two million cells were used for flow cytometry staining. FS were washed in complete media to extract cells into a 50ml conical tube and were strained through a 100 μm strainer once. CBs were inserted into 25ml serological pipette containing 20ml complete media. The CB was moved in and out of the pipette tip to dislodge the cells, while the CB was being washed in complete media to extract cells into a 50ml conical tube. Cells were then strained through a 100 μm strainer once. Both FS and CB cell suspensions were washed once with 10ml complete medium and resuspended in 100μl of complete medium and used for flow cytometric staining. Cell suspensions were placed on ice at all times to preserve cell viability. After determination of cell counts, cells were used immediately for flow cytometry. PBMCs were isolated from whole blood collected in sodium citrate tubes and isolated by density gradient centrifugation according to standard procedures as described previously [14].

### Flow cytometry

Staining on whole blood was done at room temperature while PBMCs and cells collected from CVL, FS, and CB were stained in PBS containing 2% FBS for 30 min at 4°C. In addition, leukocytes obtained from the first and second CB were separately assayed to compare CD4 T cell yield and phenotype between individual CBs. Cells were stained with fluorochrome-conjugated antibodies specific for CD45 (2D1), CD4 (OKT4), CD8 (SK1), CCR5 (2D7), CXCR4 (12G5), CD27 (M-T271), CD69 (L78) from BD Pharmingen (San Jose, CA); CD45RO (UCHL1), *FOXP3* (236A/E7) from eBioscience, (San Diego, CA); CD38 (HIT2) from Invitrogen (Grand Island, NY); and α_4_β_7_ from the NIH Nonhuman Primate Reagent Resource. Dead cells were excluded from the analysis based on staining for Live/Dead Near-IR dead cell stain from Molecular Probes, Invitrogen. Staining for *FOXP3* was performed after cells were stained for surface antigens followed by permeabilization/fixation using the *FOXP3* kit and protocol, followed by intracellular staining. Samples were acquired on a LSR Fortessa (BD Biosciences) and all cellular events were collected for mucosal samples while 500,000 events were collected for samples from blood.

### Statistical analysis

Data were analyzed using FlowJo software v X.0.7 (Tree Star, Inc., Ashland, OR) after gating out dead cells. CD45^+^, CD4^+^, and CD8^+^ T cell yields from each mucosal sampling method were summarized and compared after log transformation. T cell subset frequencies and frequency of cell-surface markers (HIV co-receptors, activation markers, and mucosal trafficking markers) were compared between each mucosal sampling method and blood. Expression of activation and mucosal trafficking markers were compared from CB and blood-derived CD45RO^+^ CCR5^+^ cells versus CD45RO^+^CCR5^-^ cells. Finally, the frequency of *FOXP3*^+^ CD4 T regulatory cells and expression of CCR5 on these cells was compared between CB- versus blood-derived CD4 T cells. For the longitudinal data, separate linear mixed effects models with random effects for participant and visit were developed for each T cell subset/ cell surface marker of interest. One thousand bootstrap repetitions were used to calculate the 95% confidence interval for correlations from repeated measures data, and statistical significance was determined by p ≤ 0.05 using correlation analysis of the first observation per participant. Wilcoxon signed rank test was used to compare paired specimens within a participant when data from only one visit was used. Statistical analyses were performed in SAS version 9.4.

## Results

We enrolled HIV negative women for up to five consecutive bimonthly visits. We excluded 5 specimens with high visible CVL blood contamination (n = 4) and limited CVL cell yields (less than 100 CD45^+^ cells, n = 1) from all subsequent analyses. Demographic, clinical, and visit characteristics of the 12 included participants contributing 33 visits are shown in **Table 1**. The median participant age was 35 years, all were African American, and the majority reported condomless sex in the last 6 months. Vaginal sex was reported during the week before 48% of study visits. Few genital tract infections were noted during study visits, but the vaginal pH was over 4.5 in over half of study visits. Any visible blood was noted at the time of collection in 6%, 41%, and 88% of CVL, FS, and CB samples, respectively.

**Table 1:** Demographic, clinical, and visit characteristics among participant visits included in the analysis.

Participant Characteristic	Number of participants (%) or median (IQR) N = 12
Age	35 (33–38)
African American race	12 (100%)
Condomless vaginal sex in the last 6 months	10 (83%)
Self-reported sexually transmitted infection^a^ in the last 6 months	3 (25%)
**Visit Characteristic**	**Number of visits (%) or median (IQR) N = 33**
Visit during follicular phase^b^	15 (46%)
Symptomatic genital infection^c^	0
Asymptomatic sexually transmitted infection	
Gonorrhea	3 (9%)
Chlamydia	3 (9%)
HSV 1/2 DNA	1 (3%)
Trichomonas	1 (3%)
pH	
Median pH (IQR)	4.7 (4.0–5.0)
pH > 4.5	17 (52%)
Self-reported vaginal sex within 1 week of study visit	14 (48%)
Semen contamination	3 (9%)
Any visible blood noted	
CVL	2 (6%)
Flocked swab	13 (41%)
Endocervical cytobrush	29 (88%)

a Gonorrhea, syphilis, chlamydia, pelvic inflammatory disease, genital herpes, genital warts, or trichomonas.

b Based on self-reported last menstrual period (follicular phase visits scheduled 7–10 days after onset of menses +/- 3 days; luteal phase visits scheduled 21–25 days after onset of menses +/- 3 days).

c Presence of purulent cervicovaginal discharge or ulcerative vaginal lesions during participant visit.

### CD4 T cell recovery is highest from endocervical cytobrushes relative to endocervical flocked swabs and cervicovaginal lavages

**Fig 1:**
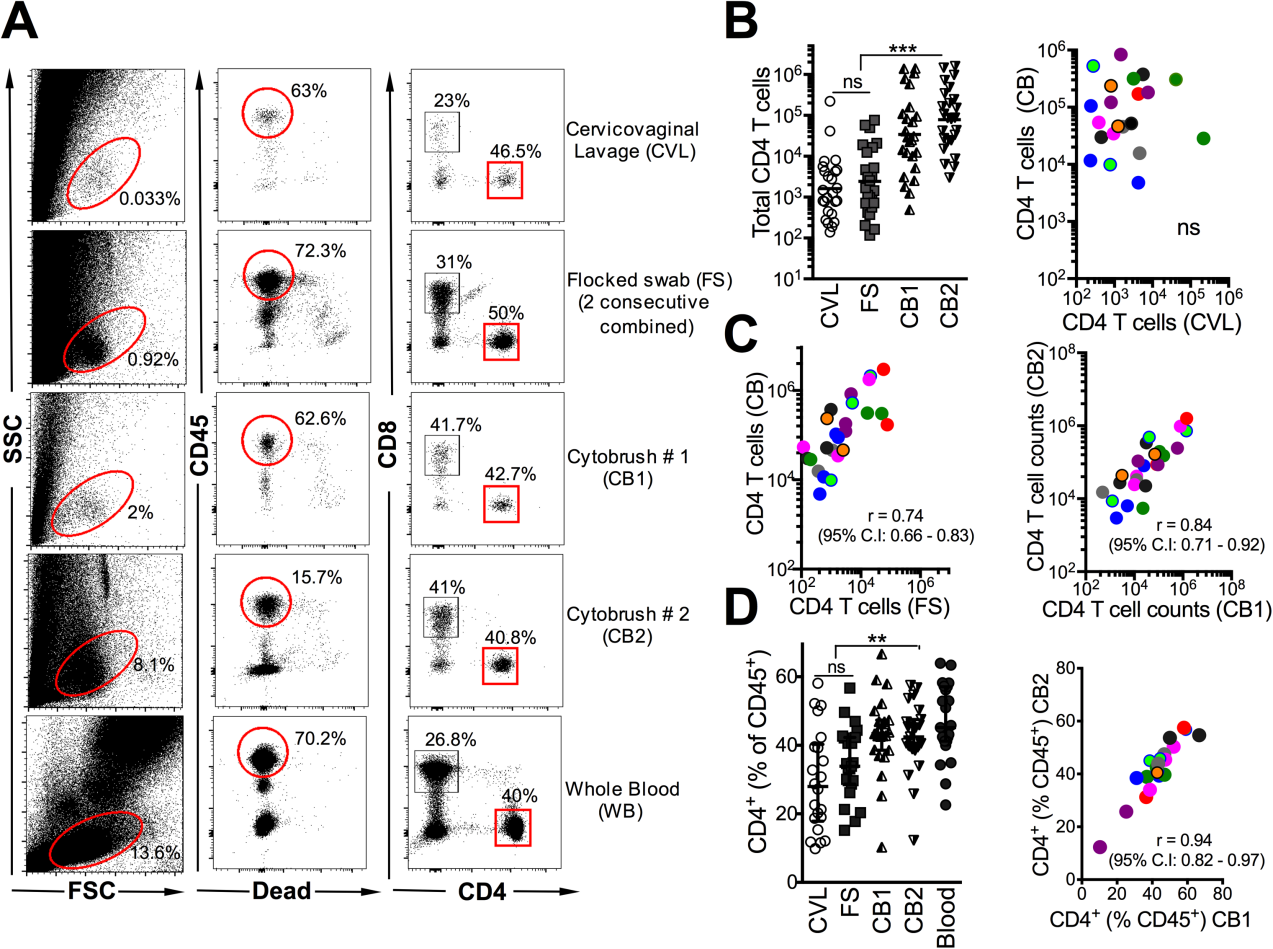
Highest CD4 T cell recovery from endocervical cytobrushes relative to endocervical flocked swabs and cervicovaginal lavages. **(A)** Example analysis of human genital mucosal specimens from 9 women sequentially sampled for cervicovaginal lavage (CVL), flocked swab (FS) followed by two consecutive cytobrushes (CB1, CB2). The cellular components were isolated by centrifugation and incubated with antibodies to identify T lymphocyte subsets at 4°C for 30 minutes. Whole blood from the same individual was stained as a positive control. Scatter plots show data for nine participants over two-three independent visits at either the follicular or the luteal phase of the menstrual cycle. Participants are color coded for correlation plots. **(B)** CD4 T cell yields significantly higher from CB sampling relative to either CVL or FS, CD4 T cell yields from CB are not associated with yields from CVL. **(C)** CD4 T cell yields from FS correlate with CB yields, and CB1 and CB2 yields strongly correlate with each other. **(D)** CD4 T cell distribution profile across sampling methods, frequency of CD4^+^ cells similar and correlated between CB1 and CB2. ***, p < 0.001; **, p < 0.01, *, p < 0.05.

**Fig 1A** shows a representative flow plot of the gating strategy used to identify CD4 T cells based on live CD45^+^ CD8^-^ leukocytes. For T cell yield analyses, we additionally excluded 8 specimens with inadequate staining for analyses and quantified the absolute number of CD45^+^, CD4^+^, and CD8^+^ cells isolated from each collection method from 9 participants over two (2 participants) to three visits (7 participants) for a total of 25 participant visits (**Table 2**). Parameters in samples excluded versus included in the analysis are shown in **S1 Table**. The median visual CB blood score (assigned 0–10 at the time of sample processing) was at least 7 for 15% and 8% for included versus excluded samples, respectively.

**Table 2:** Median (IQR) cell numbers of leukocyte subsets and ratio of CD4 to CD8 T cells in sequential cervicovaginal lavages (CVL), two consecutive flocked swabs (FS), and first and second endocervical cytobrushes (CB1 and CB2), respectively. Data are from 25 participant visits.

	CVL	FS	CB1	CB2
**CD45**^**+**^	8,526 (1,980–16,750)	5,542 (2008–38,910)	67,021 (20,116–407,211)	277,944 (46,142–796,235)
**CD4**	1,364 (402–4534)	1,697 (644–144,97)	29,501 (7,659–135,699)	85,653 (23,454–294,090)
**CD8**	782 (351–1794)	864 (291–5560)	8950 (2,505–43,116)	24031 (7,708–167,322)
**CD4:CD8**	1.7 (0.9–2.8)	1.4 (1.1–2.2)	1.7 (1.1–2.8)	1.6 (1.1–2.9)

Among all three methods tested, CB yielded the highest number of lymphocytes, CD4 T cells and CD8 T cells. The cell yield from CB2 was as good as (or in many cases was higher than) the yield from CB1. Compared to either the first or the second CB, CVL yielded markedly lower total CD45^+^ cells (median CVL 8,526; CB1 67,021; CB2 277,944; p < 0.001 for CVL vs. either CB1 or CB2), CD4^+^ cells (median CVL 1,364; CB1 29,501; CB2 85,653; p < 0.0001 for CVL vs. either CB1 or CB2), and CD8^+^ cells (median CVL 782; CB1 8,950; CB2 24,031; p < 0.01 for CVL vs. either CB1 or CB2). Cell yields from CVL were comparable to those obtained from two combined FS (FS median CD45^+^ 5,542; CD4^+^ 1,697; CD8^+^ 864. (**Fig 1B**).

To ascertain whether prior CVL or FS affected CB cell recovery, we determined correlations between cell yields from CVL, FS, and CB. Consistent with the distinct FGT compartments sampled by CVL and CB (cervicovaginal versus endocervical), we observed no association in CD4 T cell yields between the two methods (**Fig 1B**). In addition, the cellular yields from the CB samples in our study were comparable or higher than those described in other studies where CVLs were not collected prior to CB [12, 13], indicating that CVL followed by CB represents a feasible sampling strategy for assessing T cell immunobiology of the genital mucosa. In a comparison with CB samples collected without preceding FS from 6 women over 12 visits, we noted that CB yields obtained were not significantly reduced by a prior FS (**S1 Fig**). CD4 T cell yields from the FS positively correlated with yields from CB indicating similarity in compartments accessed by both procedures (**Fig 1C**). Consistently, CD4 T cell yields from the first and second CB were strongly correlated with each other (**Fig 1C**). In terms of T cell distribution, CD4 T cells (as % of CD45^+^) in CB were comparable to peripheral blood, both of which were enriched for CD4 T cells relative to CVL and FS (**Fig 1D**). We noted a strong correlation in distribution of CD4 T cells between CB1 and CB2, indicating similarity of subpopulation profiles between the two methods (**Fig 1D**). In summary, CB sampling yields higher numbers of CD4 T cells compared to FS and CVL, and two consecutive CBs provide highest recovery of CD4 T cells.

### The female genital tract mucosa is enriched with CD4 T cells expressing CCR5 and markers of activation relative to blood across all FGT sampling methods

**Fig 2:**
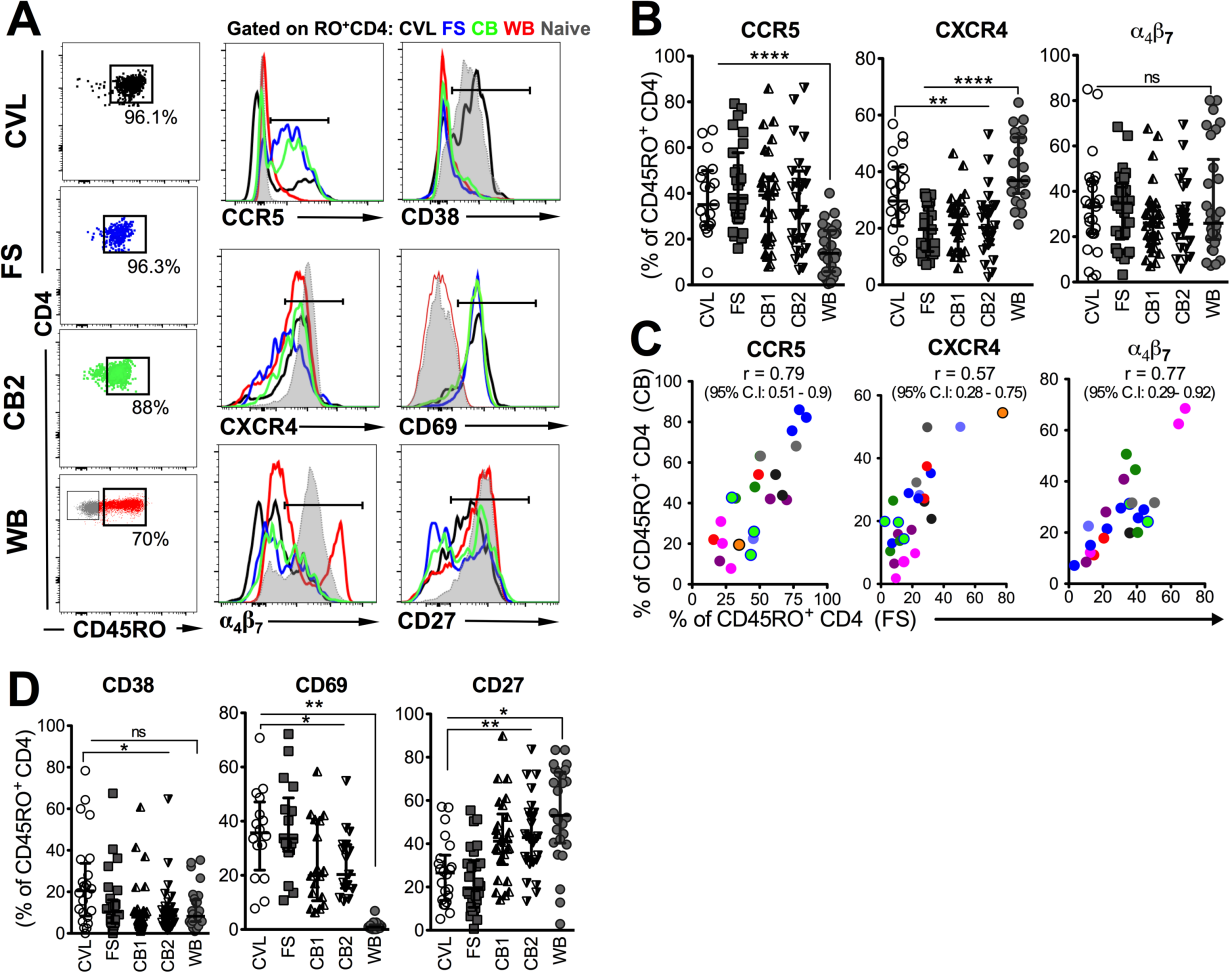
Enrichment of CD4 T cells expressing CCR5 and markers of activation in genital mucosa relative to blood. **(A)** Representative flow plots show that CD4 T cells from CVL, FS and CB are highly enriched for antigen-experienced CD4 T cells as evidenced by expression of CD45RO. Histograms show comparison of expression of CCR5, CD38, CXCR4, CD69, α_4_β_7_ and CD27 on genital and whole blood (WB) CD4 T cells. Naive CD4 T cells are overlaid in grey. **(B)** Distribution of HIV co-receptors CCR5 (n = 16 visits for CVL, 24 visits for FS, CB1, CB2, and WB), CXCR4 (n = 19 visits for CVL, 21 visits for FS, 22 visits for CB1, CB2, and WB), and expression of the integrin α_4_β_7_ (n = 22 visits for CVL, 27 visits for FS, CB1, CB2, and WB) on mucosal and blood CD4 T cells. **(C)** Correlation of CCR5, CXCR4 and α_4_β_7_ expression on FS and CB. **(D)** Distribution of activation markers CD38 (n = 21 visits for CVL, 25 visits for FS, CB1, CB2, and WB), CD69 (n = 14 visits for CVL, 15 visits for FS, CB1, CB2, and WB) and CD27 (n = 19 visits for CVL, 23 visits for FS, CB1, CB2, and WB) on mucosal and blood CD4 T cells (****, p < 0.0001; **, p < 0.01; *, p < 0.05).

We next examined CD4 T cells for expression of markers significant for HIV acquisition, HIV co-receptors, binding proteins, and markers of activation, from cells derived from each sampling method. Characteristic of enrichment of antigen-experienced T cells within effector compartments, a majority of CD4 T cells (> 85%) within the genital compartment regardless of sampling method expressed CD45RO relative to an average of 50% within peripheral blood CD4 T cells (**Fig 2A**). Therefore, to obtain an accurate representation of CD4 T cell phenotype between peripheral and mucosal compartments, phenotypic analysis was performed on memory (CD45RO^+^) CD4 T cells. We used a cut-off of 100 events within the CD45RO^+^CD4^+^ gate as criterion for further phenotypic analysis. Examination of CCR5 (histogram, **Fig 2A and 2B**) demonstrated significant enrichment of CCR5^+^ CD4 T cells in genital CD4 T cells derived from all FGT sampling methods (median CVL 35.1%; FS 37.9%; CB1 39.2%; CB2 32.7%) relative to peripheral blood (median 13.9%; p < 0.001). The frequency of CCR5^+^ memory CD4 T cells did not differ across the 3 genital sampling methods. In contrast, CXCR4 expression was significantly higher within memory CD4 T cells in the blood (median 36.8%) compared to CVL (median 29.7%; p < 0.05), FS (median 19.6%; p < 0.0001), CB1 (median 21.3%; p< 0.001), and CB2 (median 20.3%; p < 0.001). Within genital sampling methods, CXCR4 expression was significantly higher in CVL relative to FS, CB1, or CB2 (all p< 0.01). Expression of α_4_β_7_ was comparable in mucosal samples and blood with a median of 25–35% (**Fig 2B**), but we noted that per-cell expression of α_4_β_7_ was higher in blood relative to mucosal samples (**Fig 2A, histogram**) indicating possible down-regulation of α_4_β_7_ upon migration to genital mucosa. Consistent with both methods sampling endocervical areas, the expression profiles of CCR5, CXCR4, α_4_β_7_ and on CD4 T cells correlated between FS and CB samples (**Fig 2C**) but not between CVL and CB (**S2 Fig**). CD4 T cells derived from CB samples without visible blood had higher CCR5 and α_4_β_7_ expression than those with visible blood (**S3 Fig**). Together these data indicate that HIV target cells (CCR5^+^ CD4 T cells) are highly enriched in the FGT, and CD4 T cells derived from all 3 genital sampling methods express HIV binding proteins.

Next, we examined expression of markers of T cell activation across compartments. Expression of the cyclic ADP ribose hydrolase, CD38, was significantly higher in CVL-derived CD4 T cells (**Fig 2D**, median 21.5%) relative to blood (median 8.2%; p < 0.05), FS (median 9.4%; p < 0.05), and CBs (CB1 median 7.89%, CB2 8.48%; both p < 0.05). Expression of CD69, the acute marker of T cell activation and tissue retention, revealed that a greater proportion of FGT associated CD4 T cells derived from CVL (35.7%), FS (33.6%), CB1 (19.9%), and CB2 (20.3%) expressed CD69 relative to blood (0.97% p < 0.01). This attribute is consistent with the phenotype of T cells in vaginal mucosa, which are typically identified based on expression of CD69 [15]. Finally, consistent with an effector-memory phenotype, CD4 T cells within the FGT expressed significantly lower frequencies of CD27 relative to cells in the blood (median CVL, 26.5%; FS, 19.25%; CB1, 41.2%; CB2, 42.9%; blood, 53.1%). Within genital sampling methods, CVL-derived CD4 T cells expressed higher CD38, higher CD69, and lower CD27 frequencies relative to FS or CB-derived CD4 T cells. There were no differences in activation marker expression on CD4 T cells derived from CB samples without versus with visible blood (**S3 Fig**). Together, these data show enrichment of activated (CD69^+^ CD27^lo^) CD4 T cells in the FGT with higher enrichment of this effector subset in CVL samples.

When comparing expression of HIV co-receptors, binding proteins, and activation markers among CB1 samples with versus without visible blood, CD4 T cells derived from CB without visible blood had higher CCR5 and α_4_β_7_ expression than those without visible blood.

### Phenotype of HIV target CCR5^+^ CD4 T cells in genital mucosa

To better understand HIV susceptibility markers on CD4 T cells within the genital mucosa, we next performed in-depth phenotypic analyses of CCR5^-^ and CCR5^+^ memory CD4 T cells. For these analyses, we combined two sequentially collected CBs based on the preceding findings of higher cellular yield in CB samples. Because cell yields in the CCR5^-^ and CCR5^+^ subsets in the CVL and FS were insufficient for in-depth phenotypic comparisons, this analysis was performed in CB samples only. In addition, we contrasted CD4 T cell subsets in CB and blood to capture distinctive characteristics of HIV target CD4 T cells within the genital mucosa.

**Fig 3:**
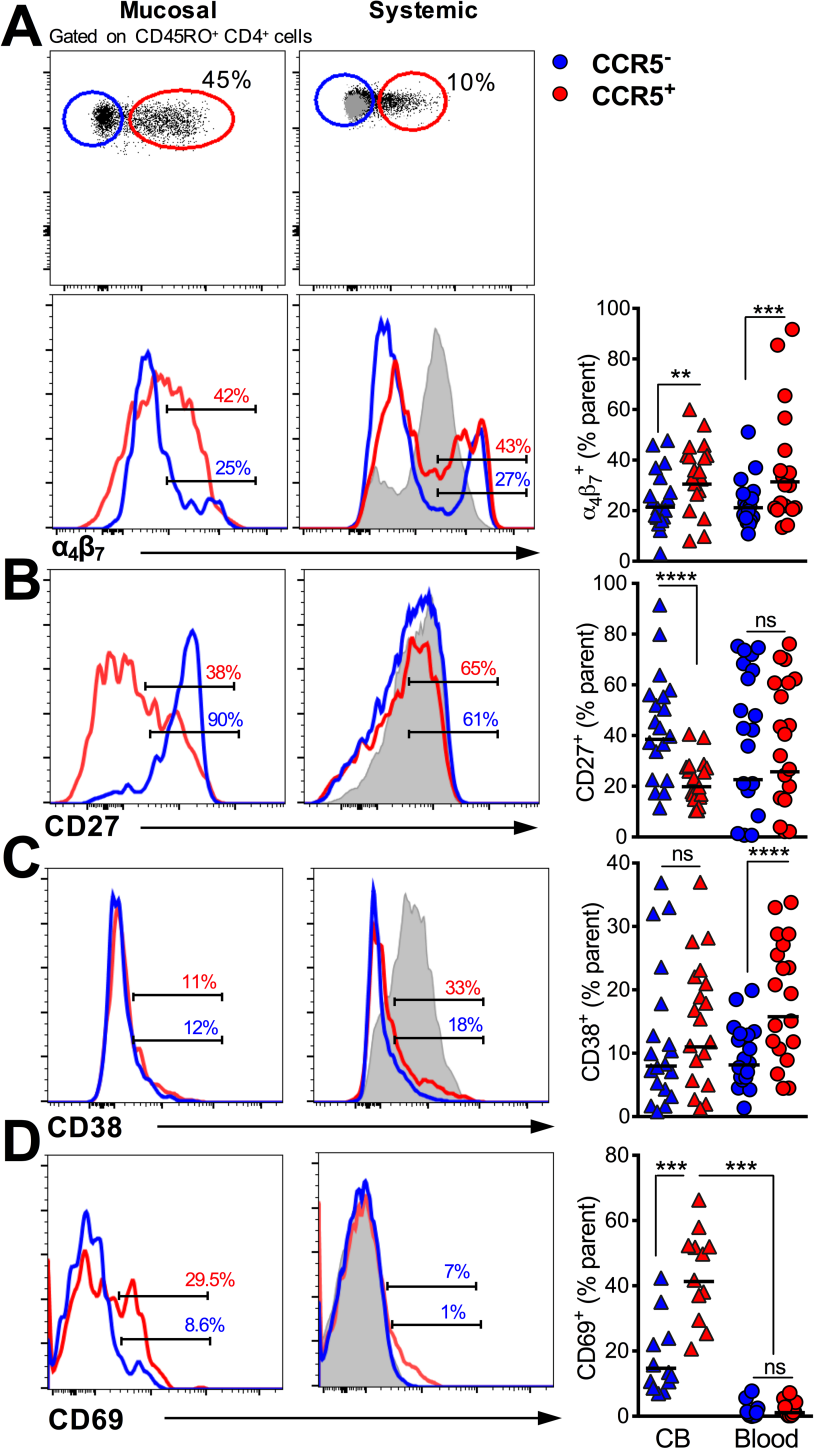
HIV target CCR5^+^ CD4 T cells in female genital mucosa display phenotypic attributes of activation and trafficking to rectal mucosa. Histograms show comparison of expression of markers between CD45RO^+^ CCR5^+^ and CCR5^-^ cells genital and whole blood CD4 T cells for **(A)** α_4_β_7_, **(B)** CD27, **(C)** CD38 and **(D**) CD69 (***, p < 0.001, n = 20 participant visits for all markers except CD69 for which n = 12 visits)

First, we evaluated whether CCR5^+^ CD4 T cells were enriched for additional markers of HIV susceptibility. Examination of CXCR4 expression revealed similar distribution profiles within CCR5^-^ and CCR5^+^ subsets in blood and FGT compartments (**S4 Fig**). On the other hand, CCR5^+^ CD4 T cells expressed higher frequencies of α_4_β_7_ in both blood and genital compartments (**Fig 3A**), indicating enhanced capacity of these cells to both bind HIV and traffic to the gut associated lymphoid tissue where HIV infection may be established.

Next, we asked whether CCR5^+^ cells demonstrated an effector phenotype and were enriched for markers of activation. CCR5^+^ CD4 T cells in the FGT, but not blood, displayed a CD27^lo^ effector phenotype [16] compared with CCR5^-^ cells, indicative of local immune activation (**Fig 3B**). While expression of CD38 was not higher in CCR5^+^ versus CCR5- CD4 T cells in the FGT (**Fig 3C)**, we found that expression of CD69, which is induced early after activation, was significantly higher in CCR5^+^ versus CCR5^-^ CD4 T cells in the FGT but had negligible expression in blood CD4 T cells regardless of CCR5 expression (**Fig 3D**). Together, these data show that CCR5 expression identifies activated effector cells within the FGT and that, among those studied, CD69 is strongly associated with CCR5 expression in the FGT.

### CD4 T regulatory cells in the genital mucosa express high levels of CCR5

Based on our data showing heighted T cell activation in the FGT, we next sought to determine the frequency and phenotype of immune regulatory CD4 T cells in the FGT and blood. For this purpose, we examined CB samples from 10 women at a single visit either at the follicular (n = 5) or the luteal phase (n = 5) of the menstrual cycle. CD4^+^ T regulatory cells (T_reg_) are a specialized CD4 T cell subset expressing the forkhead box P3 (*FOXP3*) transcription factor and maintain immune homeostasis by suppressing proliferation of effector T cells [17]. Tregs constitute 5–10% of blood CD4 T cells in otherwise healthy individuals, but little is known about whether Tregs can be detected in the lower FGT in humans and how their frequencies compare to that in blood.

**Fig 4:**
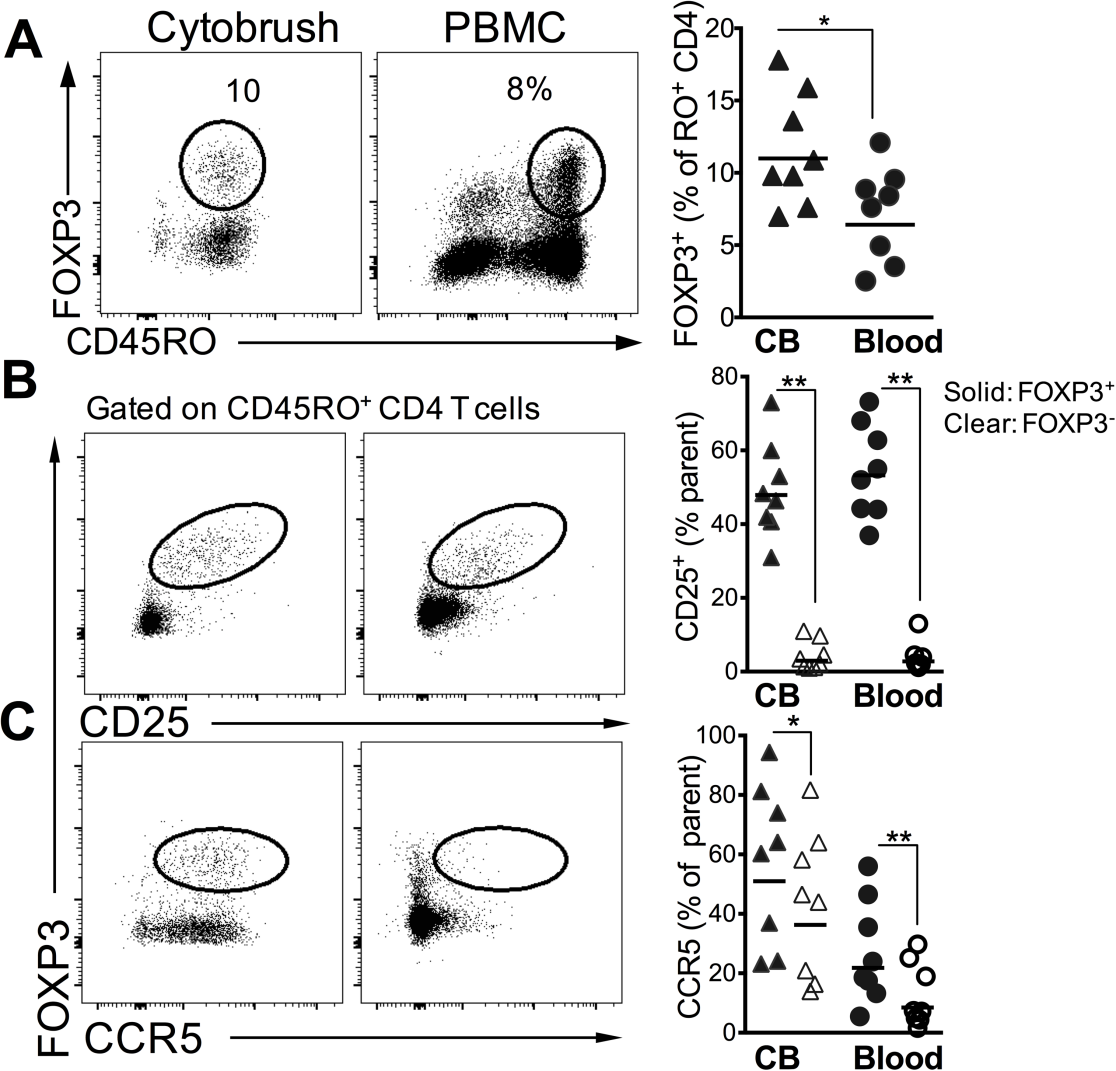
Frequency and phenotype of CD4 T Regulatory cells in the genital mucosa. **(A)** Representative flow plots showing FOXP3^+^ CD4 T cells in cytobrush and PBMCs (plots are gated on total CD4^+^ T cells). Scatter plot shows frequency of Tregs in 8 participants. (**B**) shows expression of CD25 and (**C**) shows CCR5 expression in FOXP3^+^ and FOXP3^-^ CD45RO^+^ cells (**, p < 0.01; *, p < 0.05 using a two-tailed, paired non-parametric t test

Of the 10 women sampled, we were able to detect *FOXP3* expressing CD4 T cells in discernable numbers (i.e., greater than 50 events) in CB samples from 8 women. CVL and FS samples were not tested. **Fig 4A** shows a representative flow plot of *FOXP3* expressing CD4 T cells in FGT and blood. We observed significantly higher frequency of Tregs (expressed as % of total CD45RO^+^ CD4 T cells) in CB (median 10%, range 7 to 17%) relative to peripheral blood (median 8%, range 2.5 to 12%). Consistent with phenotype of Tregs, *FOXP3*^+^ CD4 T cells were more likely to be CD25^+^, relative to non-Tregs (*FOXP3*^-^ CD45RO^+^) in both the genital mucosa and in the blood (**Fig 4B**). Because Treg numbers are associated with increased viral acquisition as observed in lymphoid tissue and mucosa in nonhuman primate models [18, 19], we also determined if Tregs preferentially expressed CCR5 in the FGT and blood. The data showed that a higher proportion of Tregs relative to non-Tregs expressed CCR5 in both the blood and FGT (**Fig 4C**). Together, these data demonstrate the presence of Treg lineage cells expressing CCR5 in the FGT in discernable numbers in the majority of participants sampled.

## Discussion

A clear understanding of immune factors that impact HIV acquisition within the FGT is important for systematic evaluation of HIV biomedical prevention strategies. This includes understanding the mucosal impact of candidate HIV PrEP drugs and microbicides, and delineating mucosal immune correlates of vaccine efficacy in HIV vaccine clinical trials for informing the rational design of HIV vaccines. Achieving this goal will require optimizing mucosal sampling strategies that can be used in longitudinal studies for accurate and effective immune profiling within the FGT. Approaches for assessment of humoral responses within the vaginal mucosa are generally well established with several studies demonstrating detection of total and HIV-specific IgG and IgA responses using vaginal swabs [20, 21]. CVL sampling is widely used for measurement of mucosal cytokines, chemokines, and antimicrobial factors [22–24] and recently, measurement of cytokines and antibodies by self-sampling using menstrual cups has been demonstrated as another tool for assessment of soluble immune factors in the genital mucosa [25]. However, there is less clear guidance regarding optimal specimen types for accurate assessment of cellular immune profiles, specifically with regards to in-depth characterization of HIV susceptibility markers on CD4 T cells.

Previous studies have used minimally-invasive methods, such as CVL [26], endocervical flocked swabs [13, 27], cytobrushes [7], and menstrual cup [28], for cellular characterization of the FGT, while others have used more invasive cervical or endometrial biopsies [7, 29] in order to attain sufficient cellular yields, but few studies exist that compare multiple minimally-invasive methods. In this study, we sought to longitudinally characterize and compare T cell yields and phenotypes using 3 minimally-invasive FGT sampling methods. Our data confirm that CD45^+^, CD4^+^, and CD8^+^ T cell recovery was highest from CB relative to FS and CVL and was consistent with one previous multicenter study which demonstrated lower yields with CVL compared to CB or cervical biopsy [12]. Our study additionally showed that FS yields correlated with CB, and FS-derived cells have similar phenotypes to those from CB with lower probability of blood contamination and may therefore provide an alternative to CB in instances where CB collection is not feasible, blood contamination is of concern, or where cells are required for other assays. The frequency of expression of HIV binding proteins CCR5, CXCR4, and α_4_β_7_ were similar among memory CD4 T cells derived from each sampling method and also correlated between FS and CB. Relative to CB, CVL-derived CD4 T cells expressed higher CD38, CD69, and lower CD27, indicative of higher activation, and potentially reflecting the different anatomic compartment (ectocervix and vagina) sampled using this method compared to FS or CB (endocervix). Our study provides the first comparison of these three minimally-invasive methods during longitudinal assessments in the same women, thereby accounting for intra-individual variability when comparing methods, and suggesting that sampling using one method did not preclude sufficient cell yields from the subsequent method.

By demonstrating the utility of minimally-invasive sampling methods for in-depth FGT investigations, our findings add to the previous literature characterizing FGT-derived CD4 T cell phenotypes and functionality in women at risk for HIV acquisition. Utilizing two consecutive CBs, we demonstrate that CCR5^+^ CD4 T cells are highly enriched within the FGT, CCR5^+^ CD4 T cells are highly activated relative to CCR5^-^ CD4 T cells in the genital mucosa. This phenotype resembles cells within the intestinal lamina propria and intraepithelial lymphocytes in humans [30]. Furthermore, expression of CD69 demonstrates recent activation and is highly consistent with the phenotype of tissue effector memory (TEM) cells, which are highly enriched in mucosa and non-lymphoid immune sites [31]. These cells rapidly produce effector cytokines upon antigen exposure [32, 33] with mouse studies showing their indispensible role in protection against HSV2 and other mucosal infections [34].

However, due to their activated state and the expression of CCR5, vaginal TEMs represent highly vulnerable HIV target cells, and studies in macaques strongly implicate their role as founder cells contributing to local viral expansion and viral dissemination [9, 35, 36]. With respect to viral dissemination, the expression of α_4_β_7_ on activated CCR5 cells in the FGT could also be highly significant. Apart from facilitating HIV binding and entry [37, 38], α_4_β_7_ directs migration of cells to gut associated lymphoid tissue [39] and could traffic HIV infected cells from the FGT to sites of active replication in the gut, thereby facilitating establishment of HIV infection [40]. Increase in frequency of α_4_β_7_^+^ CD4 T cells in the FGT is shown to enhance infection in vaginal explants [11], and blocking α_4_β_7_ protects macaques from vaginal SHIV acquisition [10]. Therefore, a better understanding of the phenotype and viral permissivity of CCR5, α_4_β_7_ co-expression on target cells in the FGT should be evaluated in future human studies and may be done so using these sampling methods. In addition, based on the relative differences in HIV transmission risk across rectal, cervico-vaginal, and penile routes, these methods could also be used to compare CD4 T cell phenotype and relative activation status across these compartments [8, 41] or understanding the relative role of macrophages versus T cells in HIV susceptibility in each of these distinct mucosal compartments [42, 43].

We demonstrated the presence of Treg lineage cells for the first time in the FGT and in the majority of participants sampled. These Treg lineage cells were more likely to express CCR5, suggesting heightened HIV susceptibility. The frequency of CD4 T regs in blood has previously been shown to correlate with estradiol levels and peak during the late follicular phase of the menstrual cycle when estrogen levels are the highest [44], and estrogen induces *FOXP3* expression *in vitro* [45], thereby supporting their potential contribution to hormonally-mediated changes in HIV susceptibility. Although the present study was not designed to capture dynamic changes in Treg frequencies within the FGT in the presence of varying endogenous or exogenous reproductive hormones, the ability to use longitudinal FGT sampling methods is critical to evaluating changes in these cells in the presence of reproductive hormones in future studies.

This study has some limitations. First, our study included African-American women who met certain HIV risk criteria and may not be generalizable to other populations. African-American women are disproportionately affected by HIV in the United States, and inclusion of African-American women in biological studies are critical to understanding HIV risk in this group. We did not, however, include women under 18 years old, and thus results cannot be extrapolated to adolescent women. Second, the small sample size was insufficient to examine the effects of multiple covariates that can affect T cell phenotype in the female genital tract or even cellular yield, including vaginal infections, the presence of semen, mucosal trauma, endogenous or exogenous hormones, and the vaginal microenvironment including the vaginal microbiome which can impact HIV risk, in part, by altering the phenotype of FGT CD4 T cells [46]. However, since all three sampling strategies were performed on each participant, the presence of these conditions would not have affected between-strategy comparisons. Third, the sequence of sampling was not randomly assigned but rather selected in the order of potential to induce trauma. While it is possible that the sequence affected cellular yields or phenotypes, correlations observed between yields and T cell markers between the second (FS) and third (CB) methods suggest that the order of sampling did not affect results. Additionally, we cannot exclude that the preceding method contributed to additional blood contamination in the subsequent method. However, correlations observed between FS (where less blood contamination was noted) and CB (where more blood contamination was noted) suggest that visible blood contamination does not likely represent peripheral blood, a finding noted in other studies[12, 47]. Furthermore, for most T cell markers, differences were not observed between CB with versus without visible blood). Finally, while we cannot completely exclude the presence of peripheral blood as a source of genital tract T cells, studies have shown that microtrauma and bleeding occurs not uncommonly during sex, so these cells are likely to enter the FGT in the setting of potential sexual exposure to HIV and as such are likely still relevant for HIV acquisition.

In conclusion, we show that all three methods may be used for cellular immune profiling in the FGT, but FS and CB sampling methods have the highest cellular yields necessary for in-depth analyses. Notably, CD4 T cells within the FGT express CCR5 and α_4_β_7_ and are highly activated, attributes which could act in concert to facilitate HIV acquisition and dissemination within the host [48]. Furthermore, we identified CD4 Treg lineage cells for the first time in the FGT and noted their high expression of CCR5, suggesting that their role in HIV acquisition should be explored in future studies. These sampling methods will allow for further investigation of strategies to reduce immune activation and/or HIV target cells at the genital mucosa as a means to enhance the efficacy of PrEP, microbicides, vaccines, and other biomedical prevention interventions, as well as to understand the potential effect of the mucosal immunity on these biomedical prevention interventions.

## Supporting information

S1 Fig Comparison of total cell yields from CB1 and CB2 either in setting of a prior flocked swab not performed (-, n = 12) versus performed (+, n = 12). (TIFF)

S2 Fig Correlation of HIV binding proteins (CCR5, CXCR4 and α4β7) expression on CB and CVL (ns, p ≥ 0.05 using correlation analysis from the first visit per participant). (TIFF)

S3 Fig Comparison of HIV binding proteins (CCR5, CXCR4, and α_4_β_7_) and activation markers (CD38, CD27) on first cytobrush (CB1)-derived CD4 T cells from specimens without (qualitative blood score < 1) versus with visible blood (qualitative score ≥ 1). CD69 analysis not performed due to small sample size (**, p < 0.01; *, p < 0.05).
(TIF)

S4 Fig Comparison of expression of HIV co-receptor CXCR4 on CD45RO^+^ CCR5^+^ and CCR5^-^ genital (CB) and whole blood CD4 T cells (ns, p ≥ 0.05 using linear mixed effects model with random effects for participant and visit). (TIFF)

S1 Table Comparison of sample parameters and cell yields between included (n = 25), excluded from all analyses (n = 5) due to high visual blood or low cellular yield, and excluded from Table 2 cell yield analyses due to inadequate staining (n = 8). (PDF)
