# Supplementary figures and images for: Characteristics of HIV target CD4 T cells collected using different sampling methods from the genital tract of HIV seronegative women
Source: PLoS One. 2017 Jun 1;12(6):e0178193. doi: 10.1371/journal.pone.0178193 (PMC5453484; doi:10.1371/journal.pone.0178193)

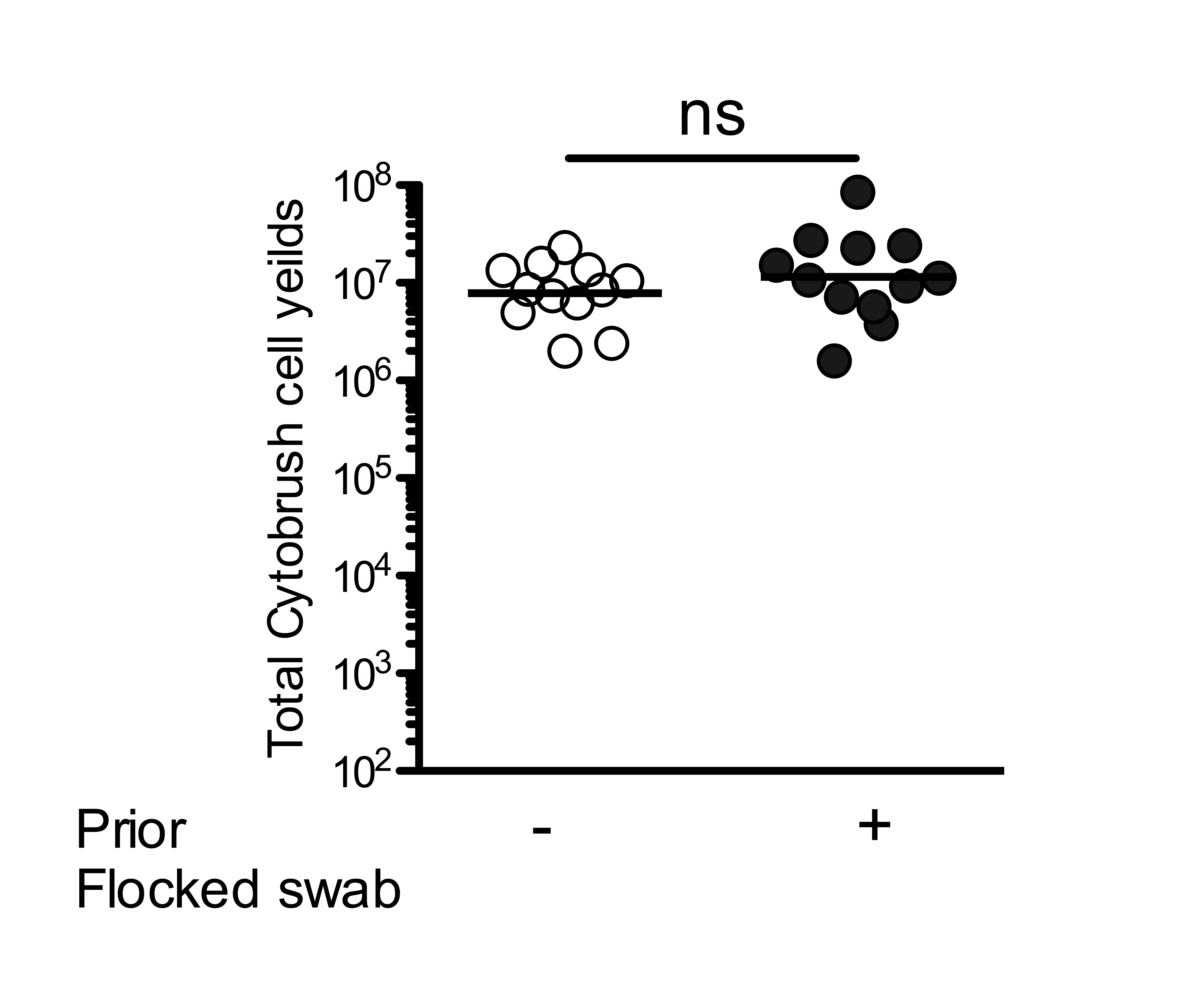

Supplement: S1 Fig — (TIFF) [file pone.0178193.s001.tiff]

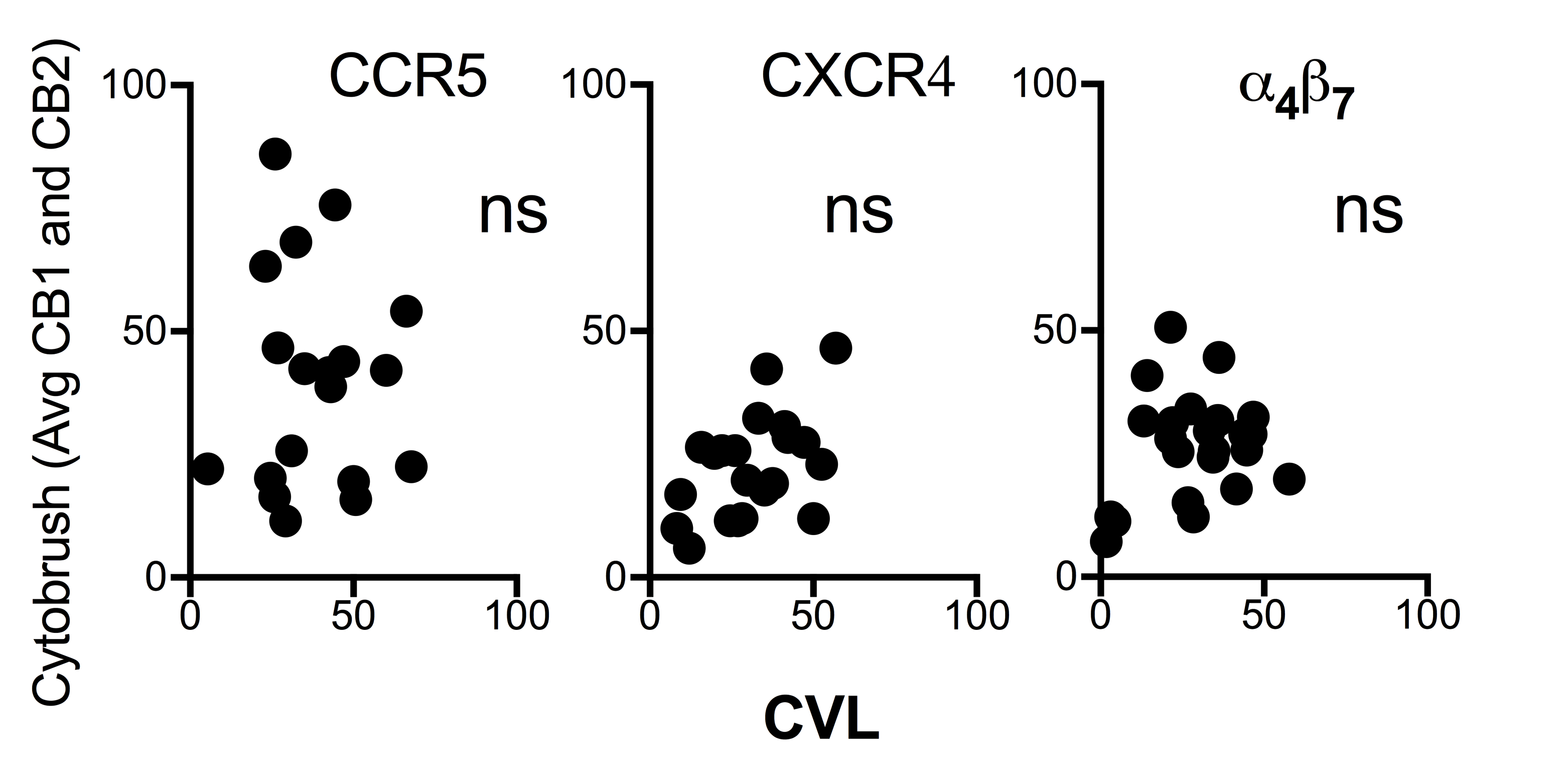

Supplement: S2 Fig — (TIFF) [file pone.0178193.s002.tiff]

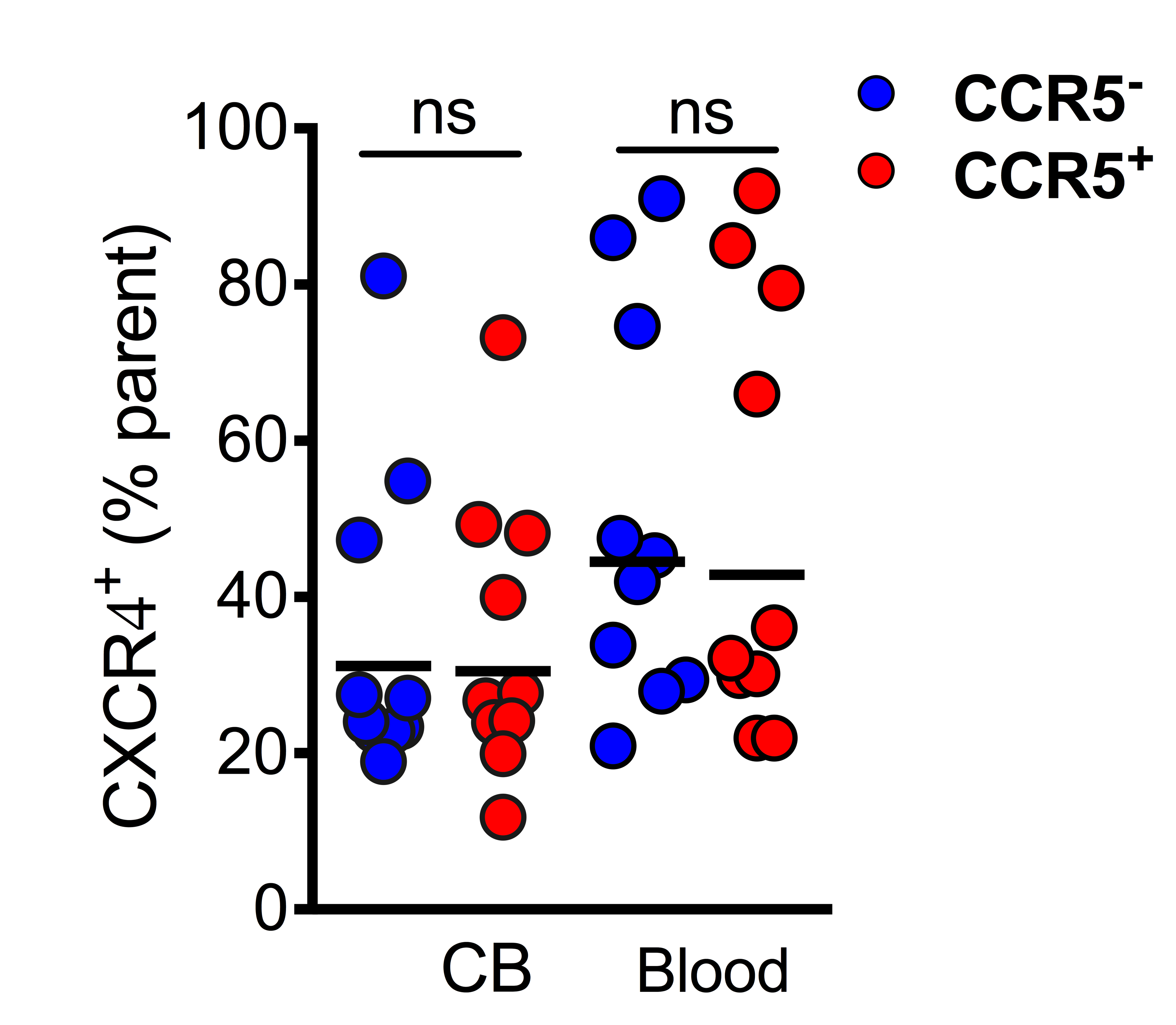

Supplement: S4 Fig — (TIFF) [file pone.0178193.s004.tiff]
