# Supplementary material for: Characteristics of HIV target CD4 T cells collected using different sampling methods from the genital tract of HIV seronegative women
Source: PLoS One. 2017 Jun 1;12(6):e0178193. doi: 10.1371/journal.pone.0178193 (PMC5453484; doi:10.1371/journal.pone.0178193)
Supplement: S1 Table — (PDF) [file pone.0178193.s005.pdf]

Supplementary Table 1. Comparison of sample parameters and cell yields between included (n=25), excluded from all analyses (n=5) due to high visual blood or low cellular yield, and excluded from Table 2 cell yield analyses due to inadequate staining (n=8).

	Included (N=25)	Excluded from all (N=5)	Excluded from Table 2 (N=8)
**CVL* blood dipstick (values 0 – 3)**	2 (0 - 3)	3 (3 - 3)	2 (1 - 3)
**First CB visual blood score ≥ 7 (values 0 – 10)**	2 (8%)	2 (40%)	0 (0%)
**Second CB visual blood score ≥ 7 (values 0 – 10)**	1 (4%)	1 (20%)	0 (0%)
**Semen (% testing positive)**	1 (4%)	1 (20%)	2 (25%)
**CVL* yield (total cells, million)**	24 (15 - 46)	24 (20 - 62)	26 (20 - 37)
**FS** yield (total cells, million)**	4.1 (2.7 – 7.5)	8.7 (3.1 - 26)	3.8 (2.8 - 7.5)
**CB*** yield (total cells, million)**	15 (9.4 - 36)	32 (25 - 120)	18 (12 - 32)

*CVL, cervicovaginal lavage; **FS, flocked swab; ***CB, cytobrush
Median (range) shown for blood dipstick and qualitative blood score parameters; median (interquartile range) shown for total cell yields and CD4 phenotype.
